# Research Progress and Future Perspectives on Photonic and Optoelectronic Devices Based on p-Type Boron-Doped Diamond/n-Type Titanium Dioxide Heterojunctions: A Mini Review

**DOI:** 10.3390/nano15131003

**Published:** 2025-06-29

**Authors:** Shunhao Ge, Dandan Sang, Changxing Li, Yarong Shi, Qinglin Wang, Dao Xiao

**Affiliations:** Key Laboratory of Quantum Materials Under Extreme Conditions in Shandong Province, School of Physics Science and Information Technology, Liaocheng University, Liaocheng 252059, Chinawangqinglin@lcu.edu.cn (Q.W.)

**Keywords:** TiO_2_, diamond, heterojunction, optoelectronic device, extreme environment

## Abstract

Titanium dioxide (TiO_2_) is a wide-bandgap semiconductor material with broad application potential, known for its excellent photocatalytic performance, high chemical stability, low cost, and non-toxicity. These properties make it highly attractive for applications in photovoltaic energy, environmental remediation, and optoelectronic devices. For instance, TiO_2_ is widely used as a photocatalyst for hydrogen production via water splitting and for degrading organic pollutants, thanks to its efficient photo-generated electron–hole separation. Additionally, TiO_2_ exhibits remarkable performance in dye-sensitized solar cells and photodetectors, providing critical support for advancements in green energy and photoelectric conversion technologies. Boron-doped diamond (BDD) is renowned for its exceptional electrical conductivity, high hardness, wide electrochemical window, and outstanding chemical inertness. These unique characteristics enable its extensive use in fields such as electrochemical analysis, electrocatalysis, sensors, and biomedicine. For example, BDD electrodes exhibit high sensitivity and stability in detecting trace chemicals and pollutants, while also demonstrating excellent performance in electrocatalytic water splitting and industrial wastewater treatment. Its chemical stability and biocompatibility make it an ideal material for biosensors and implantable devices. Research indicates that the combination of TiO_2_ nanostructures and BDD into heterostructures can exhibit unexpected optical and electrical performance and transport behavior, opening up new possibilities for photoluminescence and rectifier diode devices. However, applications based on this heterostructure still face challenges, particularly in terms of photodetector, photoelectric emitter, optical modulator, and optical fiber devices under high-temperature conditions. This article explores the potential and prospects of their combined heterostructures in the field of optoelectronic devices such as photodetector, light emitting diode (LED), memory, field effect transistor (FET) and sensing. TiO_2_/BDD heterojunction can enhance photoresponsivity and extend the spectral detection range which enables stability in high-temperature and harsh environments due to BDD’s thermal conductivity. This article proposes future research directions and prospects to facilitate the development of TiO_2_ nanostructured materials and BDD-based heterostructures, providing a foundation for enhancing photoresponsivity and extending the spectral detection range enables stability in high-temperature and high-frequency optoelectronic devices field. Further research and exploration of optoelectronic devices based on TiO_2_-BDD heterostructures hold significant importance, offering new breakthroughs and innovations for the future development of optoelectronic technology.

## 1. Introduction

With the rapid development of optoelectronic technology, there is a growing demand for high-performance optoelectronic devices. In this field, TiO_2_ and BDD are semiconductor materials that have attracted significant attention from researchers, with the former known for its outstanding photocatalytic performance and chemical stability, and the latter for its excellent conductivity, wide electrochemical window, and chemical inertness. TiO_2_, as a wide bandgap semiconductor material, exists in three crystal structures: rutile, anatase, and brookite. It has excellent optical properties, capable of absorbing visible and ultraviolet light with high photoelectric conversion efficiency, and is at the forefront of materials used in the fields of environment and energy [1]. TiO_2_ is a stable semiconductor material with high chemical and thermal stability, allowing it to operate in various environmental conditions [2]. With a wide bandgap of approximately 3.2 eV, TiO_2_ exhibits good electron transport properties and low carrier recombination rates. These characteristics make TiO_2_ widely applicable in areas such as solar cells, photocatalysis, and photodetection [3]. Different forms of TiO_2_, such as thin films (TF) [4], nanorods (NRs) [5], nanosheets (NSs) [6], and 3D nanostructures [7], have been prepared for various optoelectronic device applications including photodetectors [8], memories [9], sensors [10], light-emitting diodes (LED) [11], and field-effect transistors (FET) [12]. In recent decades, many studies have focused on growing TiO_2_ nanostructures on various materials to fabricate heterojunction optoelectronic devices, such as CuO [13], NaTaO_3_ [14], NiO [15], Al_2_O_3_ [16], ZnO [17], and BDD. BDD, a unique carbon material, exhibits extremely high thermal conductivity (approximately 22 W/cm·K), allowing effective heat dissipation and enhancing device stability and reliability [18]; it has a wide bandgap (~5.5 eV) and high electron mobility (~2000 cm^2^/V·s), resulting in excellent electron transport properties and high-speed operation capability [19]. Additionally, BDD possesses exceptional mechanical hardness, chemical inertness, and biocompatibility, enabling its stability in high-temperature, high-pressure, and corrosive environments [20,21]. These features make BDD highly promising for applications in optoelectronics, electronics, biosensing, and quantum information fields.

Considering the superior performance of n-TiO_2_ and p-BDD, it is expected to combine TiO_2_ nanostructures with BDD that can provide a method to enhance responsivity for optoelectronic applications. Due to the larger bandgap of BDD compared to TiO_2_, the combination of BDD and TiO_2_ may broaden the spectral detection range. Therefore, the n-TiO_2_/p-BDD heterojunction holds promising practical optoelectronic applications, particularly in harsh environments with high radiation, power, and temperature [1]. Recently, Liu et al. fabricated a TiO_2_/BDD heterojunction that exhibited higher responsivity than pristine TiO_2_ or BDD-based photodetectors within the ultraviolet range. This was attributed to the gradient band structure at the TiO_2_/BDD interface [21]. However, the BDD’s light response was weakened due to the absorption of ultraviolet light by the TiO_2_ TF. In this case, a novel TiO_2_/BDD ultraviolet photodetector using symmetrical Pd Schottky electrodes was developed [22], improving the BDD’s light response. It generated an extremely low dark current of 250 fA at a bias voltage of 10 V, thanks to the back-to-back Schottky contact design. Additionally, due to the accumulation of photo-generated carriers in the TiO_2_ TF, the BDD photocurrent near the cutoff wavelength exhibited differential negative conductance. As the wavelength decreased or the illumination time increased, the photo-generated carriers increased, ultimately resulting in an increase in peak photocurrent. The formation of the heterojunction can induce charge separation and carrier transport effects at the interface, thereby enhancing the photoconversion efficiency and light response speed. Furthermore, the thermal conductivity of BDD effectively dissipates heat, improving device stability and reliability [18]. These research results indicate the broad application prospects of TiO_2_-BDD heterojunctions in photodetectors, photocatalysts, optoelectronic devices, and other fields.

However, applications based on n-TiO_2_/p-BDD are limited and mainly focused on the heterojunction’s electrical transport behavior or sensor performance at room temperature (RT). The development of optical and electronic applications based on nanostructured n-TiO_2_/p-BDD heterojunctions enable them to operate in high-radiation environments (high temperature or harsh conditions) [23]. However, significant challenges still need to be addressed. Therefore, in this paper, we discussed the prospects of a variety optoelectronic devices such as photodetector, LED, memory, FET, and sensing based on n-TiO_2_ nanostructures/p-BDD heterojunctions. By analyzing the advantageous properties of TiO_2_ and BDD, as well as the characteristics of the heterojunction formed by their combination, the potential and prospects of this material combination in the field of optoelectronic devices are demonstrated [24]. Suggestions for realizing high-temperature devices based on TiO_2_/BDD composites and prospects for future research directions are introduced to facilitate the development of nanostructured materials and BDD-based heterojunctions, providing a basis for the design of high-temperature frequency optoelectronic devices. In-depth research and exploration of photoelectric devices based on TiO_2_-BDD heterojunctions can not only enhance our understanding of their optoelectronic properties and carrier transport mechanisms but also pave the way for innovative advancements in future optoelectronic technologies, driving the design and application of more efficient and multifunctional devices [25,26].

## 2. Feasible Optoelectronic Performance of TiO_2_-BDD Heterojunction

**Figure 1:**
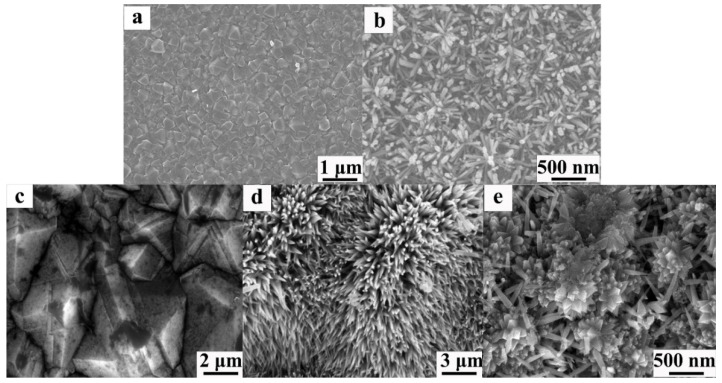
SEM images of TiO_2_ NRs grown on BDD. (**a**) BDD film; (**b**) the top views at different magnifications of TiO_2_ NRs grown on BDD with buffer layer [37]; top-view SEM images showing (**c**) the p-DBDD film, and (**d**) low- and (**e**) high-magnification views of TiO_2_ NCS on the diamond substrate [38].

In recent years, the efficient synthesis strategy of TiO_2_ NRs/nanowires (NWs)/nanotubes (NTs) prepared by hydrothermal method [27] has attracted much attention and is widely used in photocatalysis, electron field emission, photovoltaic cells, and chemical sensing [28,29,30,31,32,33,34,35]. The chemical vapor deposition (CVD) [36] BDD is an environmentally friendly p-type semiconductor, which is widely used in environmental detection, electroanalysis, and electronic devices. Yuan et al. combined a one-dimensional TiO_2_ structure with BDD to improve the performance of optoelectronic devices [37]. They reported the preparation of rutile TiO_2_ nanoparticles (NPs) on BDD films with ZnO buffer layer by hydrothermal method and investigated their morphology and properties. Figure 1a shows the SEM image of the CVD BDD film. The average grain size is about 1~2 μm top view SEM image. Figure 1b shows the uniform high-density growth of TiO_2_ NRs with diameters of 50–80 nm and an average length of 280 nm. In addition, our research group successfully synthesized TiO_2_ nanorod clusters (Ncs) structures on degenerated boron-doped diamond (DBDD) thin film substrates using the hydrothermal method [38], as shown in Figure 1c–e. The diamond thin film consists of small diamond grains with diameters ranging from 1 to 3 μm. The images reveal a rough sample surface populated with irregularly shaped nanoscale TiO_2_ Ncs. These clusters exhibit a nanorod-like crystal structure and are uniformly distributed across the diamond thin film surface.

### 2.1. UV Photodetector

In recent years, a great deal of research has been conducted on the development of deep ultraviolet (UV) and vacuum ultraviolet (VUV) light sources to meet technological needs. The development of detectors for such short wavelengths is also essential in order to match the rapid development of research and applications at short wavelengths [39,40]. Using a wide bandgap material as a photoconductive detector, a photocurrent is generated by photon absorption of generated carriers (electron–hole pairs) with an applied voltage. TiO_2_ is a wide band gap (WBG) semiconductor that can be activated by irradiating it with photons of energy greater than its bandgap; it is best known for its photocatalytic effects through increasing its absorption of visible light by narrowing its bandgap and its photocatalytic efficiency by delaying the recombination of photogenerated carriers to increase redox activity [41]. Marilou et al. prepared TiO_2_ films on soda-lime glass (SLG) substrates to construct photoconductive detectors that can be used as VUV radiation [42], but it is worth noting that BDD has a higher transmittance compared to SLG, especially in the UV spectral range. For example, in the wavelength range of 200 nm to 400 nm, the transmittance of BDD can reach more than 80%, whereas the transmittance of SLG is usually between 50% and 70%. When TiO_2_ films are used as photoconductive detectors, the high transmittance ensures that more of the UV signal can pass through the BDD substrate and into the detector structure [43]. This increases the sensitivity and photoconductivity of the detector, allowing it to receive and convert optical signals more efficiently.

**Figure 2:**
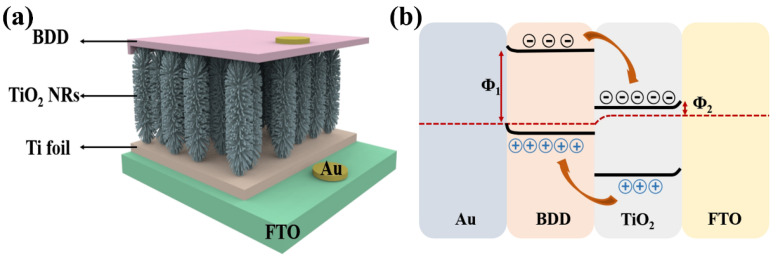
(**a**) Structure of photodetector based on n-TiO_2_/p-BDD heterojunction sandwich structure [46,52]; (**b**) a proposed physical model of energy band structure based on n-TiO_2_/p-BDD heterojunction [52].

Recently, TiO_2_ has been recognized as highly photoactive and stable under UV irradiation and has been used in the most commonly used UV sensors due to its band gap. Due to the unique property of semiconductor materials that two semiconductors with different energy band structures can improve the charge separation results, many researchers have focused on the coupling of semiconductor materials [44]. Heterojunctions of TiO_2_ with a variety of semiconductors, including CuI [45], CuMnO [46], MoS_2_ [47], and ZnO [48], have been investigated and enhanced photoelectrochemical and photovoltaic or photocatalytic properties have been observed. However, there are few p-type WBG semiconductor materials for UV detection, and most of the existing p-type materials suffer from the dilemma of low hole mobility and difficult preparation, such as p-type Ga_2_O_3_ [49] and p-type GaN [50,51]. Low mobility is a bottleneck to improving device performance (e.g., response and recovery speeds), which greatly hinders the development of p-n junction structured point-of-view detectors. However, due to the stable lattice structure of BDD and the tight atomic arrangement of the holes, electrons can be freely transported inside. BDD has a high hole mobility which is usually in the range of a few hundreds to a few thousands of cm^2^/Vs. In addition, BDD has a large bandgap width of about 5.5 eV [19], while the bandgap width of CuI is about 1.98 eV, and the bandgap width of CuMnO_2_ is between 1.5 and 2.5 eV. The larger bandgap width means that BDD is able to absorb higher energy photons, giving it better light absorption in the UV spectral range. UV photodetectors based on n-TiO_2_/p-BDD heterojunctions have great potential for application in light detection systems; based on the contributions of Mircea Nicolaescu et al. [46] and Zhang et al. [52] in the field of TiO_2_-based photodetectors, a n-TiO_2_/p-BDD composite structure can be sandwiched to form a sandwich structure between a Ti foil substrate and a Au electrode. Compared with the interdigital structure, this device structure can not only shorten the migration length of carriers, but also effectively promote carrier separation by utilizing the built-in electric field generated by the contact between the two materials, as shown in Figure 2a. And on this basis, a physical model based on energy band theory is proposed, as shown in Figure 2b. The electron affinity of TiO_2_ (3.2 eV) is lower than the work function of fluorine-doped tin oxide (FTO) (4.9 eV). The work function of Au electrode is 5.1 eV, which is much higher than that of BDD (0.2 eV). Two Schottky junctions are formed, one at the interface between TiO_2_ and FTO, and the other between BDD and Au. In addition, a type II p-n junction was formed between BDD and TiO_2_ [53,54]. When UV light irradiated the heterojunction, photogenerated electron and hole pairs were separated by the p-n junction. Free electrons were transferred to the TiO_2_ side and holes were transferred to the BDD side. When a large number of electrons are gathered on the TiO_2_ side, the Schottky barrier formed by TiO_2_ and FTO is reduced, making it easier to transfer electrons to the FTO side. Therefore, under the irradiation of UV light, the electrons are transferred from the TiO_2_ NRs to the FTO side and then recombined with the holes in the BDD side through the external circuit, and the device realizes the self-powered effect. While the schematic in Figure 2b illustrates idealized energy-band alignment, it does not account for interface defect states or surface dipoles that could significantly alter charge distribution and barrier height. Oxygen vacancies in TiO_2_ or surface terminations on BDD can introduce localized states or dipole moments, affecting carrier separation and transport. To date, first-principles simulations or density-of-states (DOS) calculations for the TiO_2_/BDD interface are lacking, representing a valuable direction for future theoretical investigation

**Figure 3:**
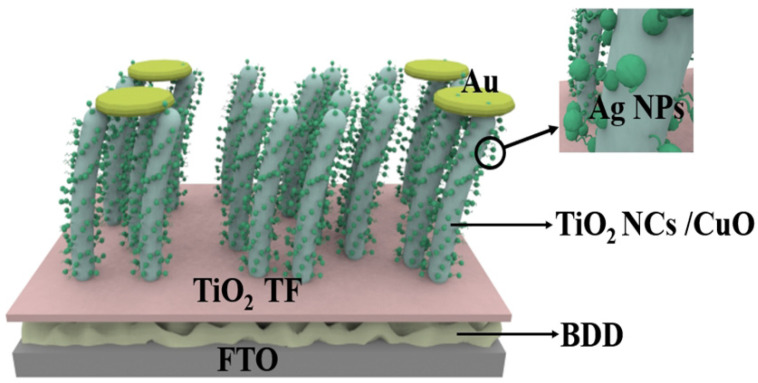
Self-powered photodetectors with p-BDD as substrate and CuO-TiO_2_ nanocomposites with attached Ag NPs [55].

Chiranjib et al. proposed an efficient RT self-powered broadband photodetector based on CuO-TiO_2_/TiO_2_/p-Si (100) heterostructure [55], which exhibits high photosensitivity and ultra-fast zero-bias response speed over a wide range of illumination (300–1100 nm). It is worth noting that although Si has abundant resources, mature fabrication technology and good semiconductor properties, it still has some disadvantages as a p-type material compared to the new material BDD. Due to the relatively high resistance of silicon, when a large current flows through the silicon device, there will be a large power loss, which may be less than ideal for some high-power applications, requiring heat dissipation and power management measures. In addition, the energy band gap (about 1.1 eV) of Si is relatively small, which leads to the Si material being more prone to thermal excitation at high temperatures, generating carriers and increasing leakage current. This may be less than ideal for some high-temperature applications and applications with high low-power requirements, in contrast to the high thermal conductivity of BDD [20], which can efficiently dissipate the heat generated in the detector. While photoconductive detectors may generate more heat under high-energy light radiation, the BDD substrate is able to conduct and dissipate the heat quickly, thus reducing heat buildup and preventing overheating from affecting the photoconductive performance. Therefore, such a self-powered photodetector based on TiO_2_/BDD can be designed as proposed by Chiranjib et al. TiO_2_ thin film layer is deposited on a p-type BDD substrate as shown in Figure 3. CuO-TiO_2_ nanocomposite is deposited on the active layer of TiO_2_ thin film and then Ag NPs are formed by thermal evaporation technique where Au is used as the metal electrode. Benefiting from the excellent thermal conductivity and thermal stability of BDD, this design could significantly enhance the detector’s high-temperature stability and thermal management performance, while also improving the sensitivity and response speed of its photoelectric response.

Despite promising responsivity and low dark current, current TiO_2_/BDD UV photodetectors face challenges such as (i) limited long-term stability under continuous UV exposure due to potential TiO_2_ degradation; (ii) narrow spectral response range unless sensitized; and (iii) lack of large-area device demonstrations. Furthermore, responsivity values (~0.1–0.3 A/W) are still lower than those achieved by commercial SiC or GaN UV photodiodes (>0.5 A/W), indicating a need for further optimization of light coupling and junction quality.

### 2.2. LEDs Application

LEDs are considered to be the main light source to replace traditional lighting in the future, with the advantages of environmental protection, low power consumption, and non-toxic materials. White light can be obtained by combining blue LED chips with yellow phosphors [56,57]. However, white LEDs often suffer from an uneven associated color temperature when projecting a light spot, i.e., the yellow ring phenomenon. In order to enhance the color temperature uniformity, scattering agents can be added to white LED encapsulants. Metal oxide particles such as TiO_2_ are widely used as potential scattering materials, among which TiO_2_ particles exhibit significant light scattering ability, improving the color temperature uniformity and light output of white LEDs. Song et al. improved the color temperature uniformity and light output of white LEDs by using silanes with different carbon chain lengths (C_3_–C_16_) to modify the surface of TiO_2_ particles to improve the optical performance and color uniformity of LED devices [58]; the dispersion of TiO_2_ increased with the increase in silane (C_3_–C_6_) carbon chain length. However, when the carbon chain length was too long (C_16_), the dispersibility of TiO_2_ was lowered instead. High luminous flux and low CCT can be achieved by adding TiO_2_ to the encapsulant, and the light performance and color uniformity of the LED device are significantly improved with the increase in TiO_2_ dispersion.

To improve the light extraction efficiency of GaN-based LEDs, TiO_2_ nanomaterials have been proposed as potential light extractors with wide bandgap, high transmittance, and moderate refractive index. Recent studies have shown that metal oxide nano- and microsphere-type nanostructures can improve the light extraction efficiency of LED chips. Transferring TiO_2_ nano- and microsphere-based monolayers onto LED chips using the icing transfer method is a feasible approach to avoid thermal damage to the chips from the high-temperature and long-time annealing process [59]. Kim et al. prepared uniformly aligned TiO_2_ nanostructured monolayers with different crystalline structures and successfully transferred them onto LED chips to improve the light extraction efficiency [60]. Experimental and simulation results confirmed the improvement of the extraction efficiency of the TiO_2_ nanostructured monolayers on LED chips. It is worth noting that BDD has a very high thermal conductivity, which is one of the best thermal conductors among known materials. LED devices generate plenty of heat during the working process, and the high thermal conductivity of BDD can effectively dissipate the heat and improve the heat dissipation effect of the LED devices, which improves the reliability and lifetime of the devices. Based on the above research we can design such a BDD-based LED while utilizing TiO_2_ nanomaterials as a potential light extractor, which will open up new ways to improve the optical performance of various optoelectronic devices (such as solar cells, photodetectors, and LED chips) [61].

**Figure 4:**
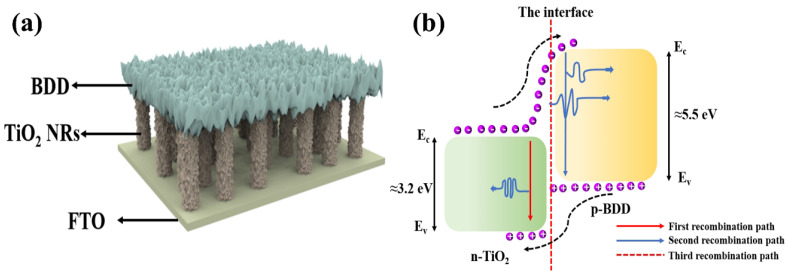
(**a**) Structure of LED device based on n-TiO_2_/p-BDD heterojunction; (**b**) LED device light-emitting mechanism schematic [63].

There are reports on the preparation of LEDs using TiO_2_ with materials such as conducting polymers and rare earth ions. However, organic material LEDs suffer from degradation problems resulting in short device lifetime [62]. To overcome this problem, El-Shaer A et al. combined n-type TiO_2_ with inorganic p-type materials such as Cu_2_O to prepare heterojunctions and act on LED light emitting devices [63]. The prepared LEDs have diode characteristics as well as high and strong electroluminescent (EL) spectra covering most of the visible range centered at 600 nm, and the EL spectra are gradually enhanced with the increase in applied voltage. In addition, BDD as a wide-band semiconductor material has a band gap of about 5.5 eV, and the physical and chemical properties of BDD can be stabilized at high temperatures due to its greatly strong and stable C-C bonds. Compared with Cu_2_O, BDD substrate can realize better thermal contact and thermal diffusion and reduce the thermal resistance to help reduce the operating temperature of the LED chip to improve the efficiency and stability of the LED [21]. Therefore, according to the research results of the above authors, it is possible to design an LED device based on n-TiO_2_/p-BDD heterojunction. In addition, compared with GaN LEDs based on Si, the use of high-temperature technology for the preparation of TiO_2_ NRs and BDD thin films is of great significance, which can realize large-scale production and provide a new reference strategy for the development of high-efficiency white light LED devices (as shown in Figure 4a). The luminescence mechanism of this device designed can be explained by energy band diagrams as shown in Figure 4b. By applying a positive external voltage, the TiO_2_ NRs inject electrons into the BDD, and the holes in the BDD move into the TiO_2_ NRs [64]. In this case, the electrons and holes can combine with each other and produce luminescence through several pathways. The first pathway is the recombination of electrons accumulated in the conduction band of the TiO_2_ NRs with the injected holes to produce photons with an energy of about 3.2 eV. The second pathway is the recombination of electrons in the conduction band (CB) of BDD with holes in the valence band (VB) of BDD, producing photons with an energy of about 5.5 eV. The third pathway is the composite of holes and electrons in the interfacial layer. In addition, defect states of TiO_2_ and BDD can also produce photons and contribute to luminescence. The energy range of photons produced by this composite is between 3.2 and 5.5 eV, thus covering the visible range. Thus, this structure provides a simple and efficient method for fabricating high-efficiency inorganic white LEDs. It is worth noting that the proposed TiO_2_-BDD heterojunction LED structures have not yet been realized or tested in practice. The discussion herein is theoretical and based on analogous systems such as TiO_2_/Cu_2_O and TiO_2_-based extractors. Further research and experimental validation are necessary to assess the actual electroluminescent performance, quantum efficiency, and emission characteristics of these devices.

Although the integration of TiO_2_ as light extractor and BDD as a heat spreader shows theoretical potential, practical TiO_2_/BDD-based LEDs remain largely unexplored. Major limitations include (i) the lack of experimental quantum yield or EL spectra data; (ii) complexity in achieving balanced carrier injection due to wide bandgap mismatch; and (iii) TiO_2_’s tendency to introduce color temperature inhomogeneity (e.g., yellow rings) unless scattering particles are well controlled. Compared to industrial GaN-based LEDs, the TiO_2_-BDD concept remains at a conceptual stage.

### 2.3. Memory Applications

**Figure 5:**
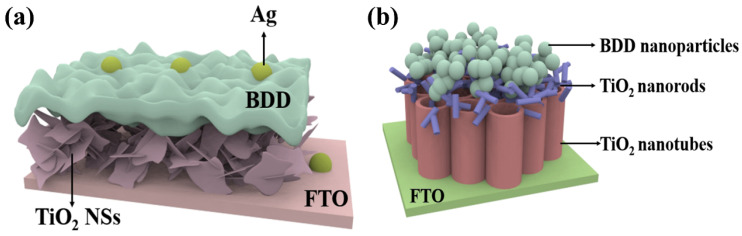
(**a**) Memory architecture based on TiO_2_ NSs/BDD/Ag NPs hybrid devices [67]; (**b**) structure of a memory device based on grafted BDD NPs TiO_2_ hybrid phase (rutile phase NTs, anatase phase NRs) [69].

Emerging two-dimensional (2D) nanomaterials such as graphene, metal oxides, chalcogenides, and organic–inorganic hybrid structures have a variety of properties such as high specific area, quantum Hall effect, and mechanical strength [65]. Among them, TiO_2_ NSs have shown excellent performance as novel 2D materials in the field of lithium-ion batteries and solar cells. When combined with organic/inorganic materials, TiO_2_ NSs can achieve desired properties such as enhanced lithium-ion diffusion and improved solar cell efficiency [66]. In addition, TiO_2_-based memristors have a wide range of applications in non-volatile memory devices. Anju Kumari et al. fabricated a hybrid device based on TiO_2_ NSs, where the pristine devices of both materials exhibited little or no resistive switching (RS) values, and the RS values of the hybridized device were improved by nearly four orders of magnitude due to the formation of p-n junctions at the NSs/polymer interface [67]. However, the economic reliability of using 2D materials remains a challenge, while the selection of suitable polymers is also an open question [68]. BDD as a p-type material can form a good energy band match with n-type TiO_2_ to promote electron transfer and carrier injection at heterojunction interfaces, in addition to being a widely available and relatively common material. It has high reliability and can be applied on an industrial scale, which makes BDD materials economically feasible and reliable for practical applications. Therefore, the TiO_2_-BDD heterojunction-based hybrid devices help to improve the response speed and read/write performance of memory devices, and provide theoretical support for further research on nanochip-based devices (as shown in Figure 5a)

TiO_2_ exists in three crystalline phases: rutile, anatase, and brookite; the properties of its nanostructures depend on the characteristics of the crystalline phases. It is shown that controlling the growth parameters and the formation of mixed-phase TiO_2_ nanostructures can achieve diverse growth behaviors [70]. Interfacial synergies between the rutile and anatase phases lead to changes in crystallinity and Ti-O bonding. Changes in lattice parameters and bond lengths lead to defects and ion polarization at the interface, affecting the surface geometry [71]. Priyanka et al. demonstrated that interfacial defects and structural features based on mixed-phase nanostructures are important for understanding the transport mechanism and performance improvement of RS devices by grafting metal NPs (Pt, Pd-Pt) [69], which further change the interfacial properties of the mixed-phase nanostructures to modulate their electronic properties. However, compared with the precious metal material Pt, which is more susceptible to chemical reactions, BDD is less susceptible to corrosion or oxidation and can better protect the long-term stability of the device structure and electrical properties which is chemically inert. In addition, BDD has excellent stability under high temperature and high pressure, and the lattice structure is not easy to change, so the modification of TiO_2_ mixed-phase memories with BDD nanostructures as grafting materials can enhance the reliability of the device and adaptability in extreme operating environments (as shown in Figure 5b).

Where the performance of the device is mainly dependent on the crystalline phase of TiO_2_, the relative yield of anatase to rutile varies depending on the grafting material and correlation due to the presence of both rutile and anatase phases in the structure formed [72] (Equations (1) and (2)).

(1) $$W_{R}=\frac{1}{1+0.8\frac{I_{A}}{I_{R}}},$$

(2) $$W_{A}=1-W_{R},$$

where *W_A_* and *W_R_* are the mass contents of anatase and rutile phases, respectively, and *I_A_* and *I_R_* are the intensities of diffraction peaks corresponding to anatase (101) and rutile (110), respectively. The grain size of nanotubes also varies with the grafting of BDD nanostructures, as calculated by the Scherrer Equation (3) [73]:

(3) $$D=\frac{k\lambda }{\beta cos\theta },$$

where *D* is the average grain size, *k* is the size-shape factor (assumed to be 1), *λ* is the x-ray wavelength (Cu *k* α source), *β* is the peak width at half the maximum arc intensity, and *θ* is the Bragg angle. *β* was obtained by Lorentzian fitting of the anatase (101) and rutile (110) peaks.

**Figure 6:**
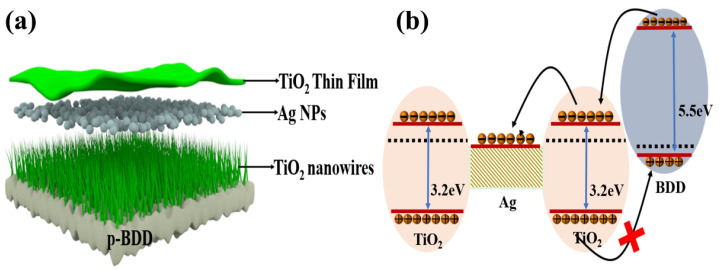
(**a**) Intercalated structure of ag nanoparticles between TiO_2_ TF and TiO_2_ NWs using BDD as substrate; (**b**) energy band diagrams of TiO_2_ NWs memory devices based on BDD, Ag NPs packages [76].

Similarly metallic NPs Ag has received much attention for its controllable growth, high thermal stability and cost-effectiveness. Ag NPs are more prone to form oxides, which may further lead to enhancement of Schottky barrier (0.3 eV) between Ag and TiO_2_ to further improve the charge storage capacity [74]. Ghosh A et al. have demonstrated the controllable Ag NP modifiers on TiO_2_ growth [75], recently Pandey A K et al. have built on the work of Ghosh A et al. by depositing Ag NPs on TiO_2_ NPs using a simple, well-controlled and cost-effective technique [76]. This novel structure sandwiches Ag NP between TiO_2_ NWs and thin film, which allows for the introduction of an intermediate state between the TiO_2_ thereby creating a charge trapping center and injecting a higher charge, thereby improving the characteristics of non-volatile memory storage [76]. It is worth noting that the design is based on a p-type Si substrate. BDD as a wide bandgap semiconductor material has a lattice structure with excellent thermal stability at high temperatures. Even under the extreme environment of 700 °C, BDD is able to maintain its lattice integrity and electrical stability, far exceeding that of Si-based semiconductors; BDD’s bandgap is as high as 5.45 eV, which is five times higher than that of typical semiconducting silicon which gives it an extremely high electrical insulation to effectively inhibit leakage current, and maintain excellent insulation and device stability under high pressure and high power. Therefore, the replacement of Si substrate by BDD substrate (as shown in Figure 6a) may provide a more solid theoretical foundation for this memory device to work under high temperature and high pressure.

During the positive voltage scanning process, electrons are easily injected from the p-type BDD substrate to the interface between Ag NPs and TiO_2_, forming a shallow trap energy level for trapped electrons. With the increase in positive voltage, the number of trapped electrons in Ag NPs continues to increase, and these electrons can lead to the upper electrode through the trap-assisted tunneling effect in the TiO_2_ layer, resulting in charge collection during the programming process [77]. During the negative voltage scanning process, the holes stored in the Ag NPs are difficult to be removed due to the large offset of the VB between the p-type BDD and TiO_2_. With the increase in the negative voltage, more holes are trapped in the Ag NPs, which further increases the negative voltage offset of the device and thus improves the storage performance. In conclusion, the memory device utilizes the p-BDD/n-TiO_2_ heterostructure and the charge trapping property of Ag NPs to realize reversible charge injection and collection, and exhibits good storage function (as shown in Figure 6b).

In TiO_2_-BDD-based resistive memory, the key advantages stem from interface control and thermal stability. However, the reproducibility and endurance (typically <10^4^ cycles) lag behind established ReRAM technologies (10^6^–10^9^ cycles). Moreover, the influence of BDD’s surface roughness and diamond grain boundaries on switching uniformity remains insufficiently studied.

### 2.4. FETs Applications

Dielectric materials in high-performance FETs play an important role in realizing low-voltage and high-performance FETs [78,79,80]. Among the common dielectric materials, TiO_2_ is a low-cost option with a high relative dielectric constant (*k*). However, due to its narrow bandgap, TiO_2_-based FETs usually suffer from large leakage current problems [81]. In order to improve the performance of TiO_2_ and reduce the leakage current, researchers have used several methods, such as the introduction of bilayer dielectrics, doping of inorganic materials, and compositing with organic materials [82]. Yang et al. obtained TiO_2_: polymers (e.g., polyvinylpyrrolidone, PVP) composite dielectrics with high relative permittivity and low leakage current density by introducing organic PVP compositely modified with TiO_2_. The composite material combines the advantages of inorganic and organic materials, and has excellent properties such as high transmittance, low surface roughness, and amorphous characteristics [83]. Metal-insulator-metal (MIM) devices with excellent dielectric properties were prepared by using TiO_2_ and PVP composite dielectric as an insulating layer. This inorganic–organic composite dielectric has potential applications in the field of low energy consumption and high-performance printed electronics.

**Figure 7:**
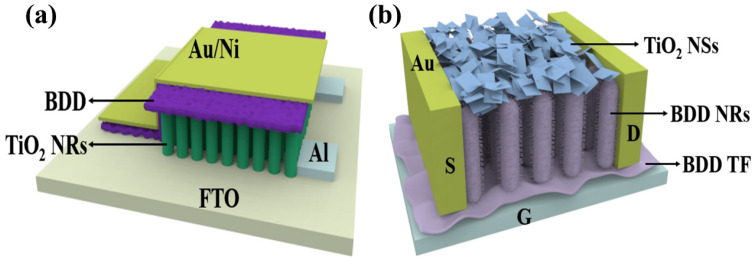
(**a**) Structure of an FET designed with TiO_2_ as substrate and BDD as gate dielectric layer [87]; (**b**) schematic of back-gated BDD NRs phototransistors deposited by TiO_2_ NSs [89].

Metal oxide FETs have attracted attention for their high optical transparency, high carrier mobility, and ease of production. TiO_2_ FETs utilizing TiO_2_ as the channel material exhibit performance comparable to that of conventional Si-based FETs, with the advantages of low cost and abundant resources [84]. Zhang et al. significantly improved the performance of TiO_2_ FETs by pre-annealing them with oxygen and nitrogen during the device fabrication process [85]. The oxygen pre-annealed TiO_2_ FETs have higher mobility (*μ*), higher *I_ON_/I_OFF_* ratio, and lower subthreshold swing (SS) than the nitrogen pre-annealed FETs. Oxygen annealing provides an effective way to improve the performance of metal oxide FETs, in which oxygen vacancies play an important role in the electrical properties. This study demonstrates the feasibility of TiO_2_-based FETs and the fact that controlled oxygen and nitrogen annealing techniques can be used as a complementary method for realizing different modes (enhancement/depletion) of FETs, providing greater flexibility for TiO_2_-based large-scale electronics and logic circuit applications. Building on this foundation, the team continued its research in the area of the effect of the gate dielectric on the electrical properties of FETs. Theoretically, reducing the dielectric thickness can significantly improve device performance as it helps to enhance electrostatic control of the gate [86]. However, in practice, achieving nanoscale dielectric thickness scaling faces a number of challenges that place stringent demands on the quality of the dielectric material. These challenges mainly involve increased leakage currents, unstable threshold voltages and reduced channel mobility, as well as changes in interfacial properties. In order to fully investigate the effect of dielectric thickness on FET performance, the team systematically investigated the effect of ZrO_2_ gate dielectric thickness on the electrical characteristics of TiO_2_ FETs [87]. By performing exhaustive electrical measurements and employing different thicknesses of ZrO_2_ dielectrics, they revealed the correlation behaviors between dielectric thickness and leakage current, oxide capacitance, oxide charge, and interface traps [88]. The results show that ZrO_2_ possesses excellent thickness scalability and provides reliable dielectric properties even at ultra-thin physical thickness of only 5 nm. TiO_2_ FETs prepared using 5 nm-thick ZrO_2_ dielectrics exhibit excellent electrical properties, including a high on/off current ratio (*I*_on_*/I*_off_) of 7.7 × 10^8^ at very low voltage (2 V), a nearly ideal subthreshold slope (72 mV/dec), and an electron mobility (*I*_on_*/I*_off_) of up to 5.74 cm^2^·V^−1^·s^−1^ electron mobility (*μ*_eff_). The results of this study are important in revealing the quality and scalability of the gate dielectric and can provide lessons for similar studies of other material systems. However, it is worth noting that further reduction in thickness leads to the breakdown of the FET, reflecting the physical limitations of ZrO_2_ ultrathin dielectric materials. In contrast, BDD is able to resist stresses and deformations, reducing device failures caused by mechanical stresses due to the extremely high hardness and strength. BDD also has a high resistivity (10^12^ Ω·cm) and excellent electron transport properties, which help to reduce the current leakage of the gate material, improving the conductivity and response speed of FETs and realizing higher operating frequency and lower power consumption. Therefore, it is possible to design TiO_2_-based metal FETs with BDD gate material (as shown in Figure 7a), and the large CB offset between BDD and TiO_2_ may be a potential physical reason for the excellent thickness scalability of BDD [21]. Based on the feasibility of TiO_2_-based FETs for practical applications demonstrated by Zhang et al., the modulation of BDD in the gate material may provide theoretical support for low-power requirements and transistor devices for applications in extreme environments. The advantages of BDD material over traditional ZrO_2_ material in terms of device performance, reliability, and application prospect provide new ideas and possibilities for the development of new high-performance FETs devices.

The BDD/TiO_2_ interfaces have attracted much attention in recent years because of the need to gate ultrathin TiO_2_ through a dielectric oxide layer in various devices [90]. In addition, BDD can be used to protect and encapsulate TiO_2_-based devices from damage by harsh environments [91,92]. When oxidation occurs at the TiO_2_ interface, it leads to energy band alignment and charge transfer. Numerous studies have been conducted to explore the interaction between pure carbon materials and TiO_2_. For example, combining monolayer/few-layer BDD, graphene, or carbon NTs with TiO_2_ can help to achieve charge separation at the interface, which can alter the intensity of photoluminescence (PL) [93,94] or enhance the photocatalytic effect [95]. These modulations can be further carried out by external gate voltage or light illumination to achieve electronically or optically controlled response of the device. Preparation of 2D BDD NRs structures can be carried out using a variety of methods, including plasma post-treatment of carbon NTs, reactive etching, and high-temperature and high-pressure methods for transferring fullerenes onto BDD NRs. Therefore, the electrical and photoelectric properties of TiO_2_ can be effectively modulated based on the results of Liu et al. [89] The 2D BDD NRs structure can be used as a template for the horizontal growth of ultrathin TiO_2_ NSs, and the BDD NRs structure can guide the ultrathin TiO_2_ NSs to be ordered in the horizontal direction, as shown in Figure 7b. This design provides valuable insights into the interactions at the BDD/TiO_2_ interface and offers a new avenue for the development of novel optoelectronic devices based on 2D pure carbon materials/TiO_2_ hybrids.

While TiO_2_-BDD offers low leakage current and thermal robustness, the field-effect mobility remains modest compared to IGZO (>10 cm^2^/V·s) or 2D materials like MoS_2_ (>100 cm^2^/V·s). Also, scalable low-temperature fabrication compatible with CMOS remains a barrier. The mechanical hardness of BDD could pose integration challenges with flexible substrates.

### 2.5. Sensing Applications

Gas sensors are devices used to detect the concentration of different gases in the environment, and they find wide applications in areas such as environmental protection, industrial safety, medical diagnostics, and biochemical analysis [96,97]. Metal oxide semiconductors (MOSs) are commonly used as sensing materials, and their surface chemical properties play a crucial role in sensing performance. Researchers are devoted to improving the performance of MOSs sensing materials. One key challenge is to increase the number of active sites to enhance the sensitivity and selectivity of the sensor. To address this, researchers have employed various strategies. One commonly used strategy is selective exposure to high-energy crystal facets to increase the number of active sites and the adsorption of oxygen [98]. Through this approach, the sensing material’s response to the target gas can be improved. Another strategy is the preparation of nanocomposite materials, where MOSs is combined with other materials to form composite structures. This method can increase the specific surface area of the sensing material and introduce suitable catalytic particles, further enhancing sensing performance. For example, constructing heterogeneous interfaces with a large specific surface area can enhance the sensitivity and stability of the sensor [99].

In addition to optimizing the composition of materials, researchers have recently turned their attention to regulating the crystal phase structure of sensing materials to improve their sensing performance. By adjusting the crystal phase, the electronic structure and surface activity of the material can be modulated, thereby altering the sensor’s response to different gases. For example, researchers have found that changing the proportion of different crystal phases in MOSs can significantly affect the sensor’s response to specific gases [100,101,102]. This discovery provides a new approach to modulating the crystal phase structure to enhance sensing performance. Furthermore, TiO_2_ is a commonly used sensing material that exhibits three common crystal phases: rutile, anatase, and brookite. Different crystal phases of TiO_2_ exhibit distinct sensing characteristics, leading to in-depth research by Cao et al. [103] By regulating the crystal phase structure and preparing TiO_2_ materials with specific crystal phases, the researchers demonstrated that sensors based on rutile-type TiO_2_ NRs exhibited high sensitivity, rapid response times, excellent stability, and good selectivity, displaying a high response capability to various volatile organic compounds (VOCs) [104]. The adjustment of crystal phase structure played a critical role in improving sensing performance. By modulating the band structure, rutile-type TiO_2_ NRs could effectively adsorb active oxygen ions on the material’s surface [105], thereby enhancing the sensor’s response capability. This research outcome provides important insights for further optimizing the gas-sensing performance of MOSs. In TiO_2_/BDD-based sensors, oxygen vacancies are located in the TiO_2_ lattice and serve as active sites for gas molecule adsorption and electron exchange. TiO_2_ is the primary gas-sensitive material interacting with analyte gases, while BDD acts as a chemically inert yet highly conductive substrate that facilitates rapid charge transport, thermal dissipation, and structural stability under harsh environments. By adjusting the crystal phase structure, more efficient and accurate gas detection and analysis can be achieved. The research conducted by Cao et al. highlights the extensive potential application of TiO_2_ in the field of sensors. Particularly for gas sensors, TiO_2_ nanostructures demonstrate excellent performance, showcasing the advantages of TiO_2_ as a sensing material. The high sensitivity and selectivity of TiO_2_ nanostructures towards VOCs such as acetone demonstrate that TiO_2_ can effectively detect and differentiate different gas components. This is of significant importance in areas such as environmental monitoring, industrial safety, and biochemical analysis. The fast response time and outstanding stability of TiO_2_ nanostructures enable real-time monitoring of gas concentration changes while maintaining long-term reliable performance. This is crucial for real-time monitoring and early warning systems, ensuring timely measures are taken to ensure safety and environmental protection. By regulating the crystal phase structure of TiO_2_, especially optimizing the proportion of crystal phases, the performance of TiO_2_ sensors can be further improved. The successful application of TiO_2_ nanostructures demonstrates that adjusting the crystal phase can alter the material’s electronic structure and surface activity, thereby enhancing the sensitivity and response capability of the sensor.

**Figure 8:**
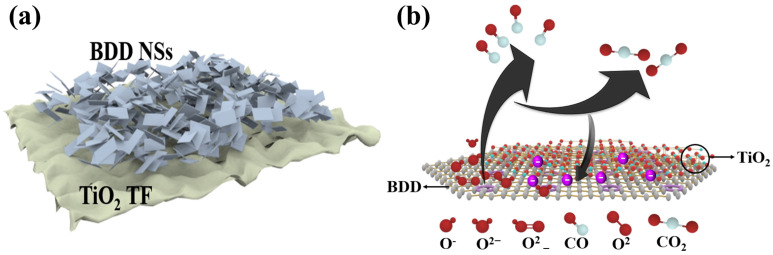
(**a**) CO gas sensor based on TiO_2_ TF/BDD NSs heterojunction; (**b**) sensing mechanism of surface charge in TiO_2_ TF /BDD NSs [108].

Carbon monoxide (CO) is a highly harmful substance produced by the incomplete combustion of carbon-containing materials [106]. It can bind to red blood cells, impair oxygen supply, and in severe cases, even lead to death. Due to its detrimental effects on human health and the environment, CO is considered a significant contributor to air pollution, with a typical exposure limit of 50 ppm. To effectively monitor and manage combustion devices fueled by carbon-containing substances, there is a need to develop highly responsive and selective CO gas sensors. Metal oxide CO sensors are advantageous due to their simplicity, low cost, and versatile applications [107]. By combining hydrothermal synthesis and flame annealing techniques, Chen et al. successfully prepared porous TiO_2_/CeO_2_ NSs with varying amounts of TiO_2_ coupling [108]. Through bandgap engineering of the TiO_2_ and CeO_2_ heterostructures, the composite material exhibited a narrowed bandgap and improved electron transfer efficiency, resulting in excellent gas-sensing performance of TiO_2_/CeO_2_ NSs. Experimental results showed that the response value of TiO_2_/CeO_2_ NSs was 9.78 times higher than that of pure TiO_2_. Additionally, TiO_2_/CeO_2_ NSs demonstrated ultra-fast response and recovery times. Moreover, the sensor exhibited good selectivity towards CO and could detect CO concentrations ranging from 500 ppm to 5000 ppm. These outstanding properties make TiO_2_/CeO_2_ NSs sensors hold great potential for rapid CO monitoring. However, there has been limited research on CO gas sensing using CeO_2_, mainly focusing on nanostructured films with longer response/recovery times and narrower detection ranges [109,110]. On the other hand, BDD as a special semiconductor material possesses excellent electrochemical stability and controllable surface charge transfer properties. This enables TiO_2_/BDD NSs to quickly recover to the baseline state. This rapid recovery capability allows the sensor to exhibit good stability in applications requiring continuous monitoring and multiple measurements [111]. BDD also has extremely fast electron transfer speed and high carrier mobility which accelerates the electron response and transfer process when combined with the heterostructure formed with TiO_2_. This feature makes TiO_2_/BDD sensors exhibit fast response in gas detection. Reported TiO_2_-based sensors exhibit sensitivities enhanced by nearly an order of magnitude when combined with heterostructures and fast response times typically below 10 s for CO or VOC detection. The fast response is defined as the time taken for the sensor to reach 90% of the final signal change upon gas exposure. Based on the research by Chen et al., a CO gas sensor based on TiO_2_/BDD NSs heterostructure can be designed (as shown in Figure 8a).

The calculation formula for the gas sensitivity response value (*S*) is as follows.

(4) $$S=\frac{\left|R_{a}-R_{g}\right|}{R_{g}}\times 100\%$$

In Equation (4), *R_a_* and *R_g_* represent the resistances of the device in air and the target gas, respectively. Furthermore, we envisioned the working principle of this heterojunction when exposed to a CO environment (as shown in Figure 8b) [108]. When TiO_2_/BDD NSs are exposed to air, oxygen molecules will adsorb onto the surface of TiO_2_/BDD NSs, capturing electrons from the conduction band. This leads to an increase in resistance and the expansion of the space charge region in TiO_2_/BDD NSs. Additionally, different adsorption states of oxygen (such as O, O^2−^) are formed depending on the temperature. When TiO_2_/BDD NSs are exposed to a CO atmosphere, CO reacts with the surface-adsorbed oxygen, reducing CO to CO_2_ and releasing electrons back into the conduction band of TiO_2_/BDD NSs. Therefore, by monitoring the resistance changes of TiO_2_/BDD NSs in different atmospheres, the concentration and type of gas can be determined. In the gas-sensing process, oxygen vacancies (V_O_) play an important role in TiO_2_/BDD NSs. As defects, V_O_ possess adsorption capabilities, facilitating the adsorption of more oxygen molecules onto the surface of TiO_2_/BDD NSs and further dissociating into adsorbed oxygen. Additionally, V_O_ can also promote the adsorption of target gas molecules and react with the adsorbed oxygen. Thus, V_O_ plays a positive role in the gas-sensing process [112]. Therefore, TiO_2_/BDD NSs utilize resistance changes to determine the concentration and type of gas when exposed to different atmospheres. Simultaneously, the adsorption and reaction of oxygen molecules, as well as the presence of oxygen vacancies, are crucial for the gas-sensing performance of TiO_2_/BDD NSs. This understanding helps further optimize the design and performance of TiO_2_/BDD NSs sensors, providing a more reliable and efficient solution for gas detection.

The advantage of core–shell heterostructures lies in their ability to achieve complete interface contact between two components, thereby maximizing the efficiency of interactions between materials [113]. This unique optoelectronic property makes it an ideal choice for constructing highly efficient photoelectrochemical (PEC) biosensors [114]. Additionally, the shell in the core–shell structure acts as a dispersant and intermediate layer, effectively suppressing the aggregation of active substances and improving the stability and performance of the sensor. One-dimensional (1D) core/shell structures have significant advantages in optimizing interface charge transfer, making them highly promising for PEC biosensors [115]. Taking BDD as an example, it is a special p-type semiconductor suitable for building II-type heterojunctions with TiO_2_. Furthermore, p-type semiconductor BDD has strong adsorption capabilities between electrode materials and enzymes which is advantageous for interface stability and catalytic efficiency. The utilization of BDD as the shell component not only promotes the separation of photogenerated charge carriers but also effectively prevents the decomposition of enzymes by high-oxidation holes generated in the TiO_2_ core under illumination. This protective effect is crucial for maintaining enzyme activity and long-term stability of the sensor.

By introducing the BDD shell, a high-quality interface structure can be formed, enabling efficient transfer and separation of photoelectrons. The BDD shell plays a key role in the interface structure through II-type band edge alignment and enhanced chemical bonding interactions, facilitating the ordered transfer of photoelectrons. Photoelectrons can smoothly transfer from the conduction band of the BDD shell to the CB of the TiO_2_ NRs core [116]. Simultaneously, photogenerated holes in the TiO_2_ NRs core can effectively transfer from the VB to the BDD shell. This transfer of photoelectrons and photogenerated holes facilitates the efficient separation of photoelectrons and holes between the TiO_2_ core and BDD shell. Through this optimized interface structure and photoelectron separation, 1D core/shell structures exhibit higher photoelectric response capability and photocatalytic activity in PEC enzyme biosensors. Additionally, BDD possesses excellent chemical stability and corrosion resistance, making it an ideal shell material that can resist acid-base corrosion and oxidation–reduction reactions, thus protecting the internal TiO_2_ core material from adverse effects in the environment [117]. This chemical corrosion resistance enables PEC biosensors based on 1D core/shell structures to have a longer lifespan and better stability in complex biological samples or environments. The high conductivity and low electron recombination ability of BDD also contribute positively to the transfer and separation of photoelectrons.

**Figure 9:**
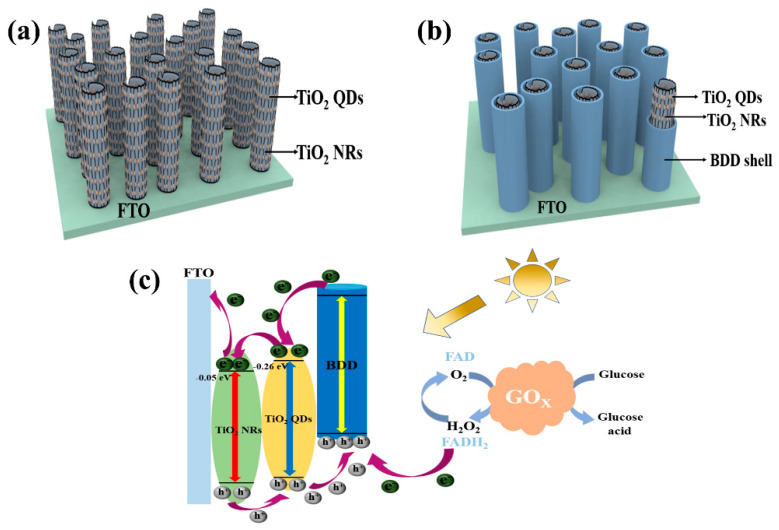
(**a**,**b**) 1D core–shell structure with TiO_2_ NRs as the core and BDD as the shell and attached TiO_2_ QDs acting on sensor devices; (**c**) mechanism of operation of the intended design of the sensor [122]. Although both TiO_2_ and BDD are wide bandgap materials, the use of TiO_2_ quantum dots introduces sub-bandgap absorption due to quantum confinement and defect states, allowing visible light excitation. BDD may also contribute to visible-light absorption via π–π transitions or boron-induced acceptor levels. In the proposed heterostructure, TiO_2_ QDs are the primary absorber and electron generator under visible light, while BDD efficiently collects holes and transports them to the enzymatic interface. (**b**) illustrates the working mechanism of the designed biosensor. BDD is capable of absorbing light and inducing π-π* transitions under visible light. Excited-state electrons transition from the highest occupied molecular orbital of BDD to the lowest unoccupied molecular orbital. The conduction band potentials of TiO_2_ QDs and TiO_2_ NRs are approximately −0.26 eV and −0.05 eV, respectively. Therefore, in this process, photoelectrons first transfer to the CB of TiO_2_ QDs and then to the CB of TiO_2_ NRs [123], ultimately reaching the FTO substrate. TiO_2_ QDs provide more charge carriers and higher charge transfer efficiency, suppressing the recombination of photoelectrons and holes, thereby enhancing the PEC performance of the material [124]. The holes generated in BDD are transferred to glucose oxidase (GO_X_), facilitating the oxidation of glucose to gluconic acid by GO_X_. The presence of BDD effectively prevents enzyme deactivation and improves the biocompatibility of the electrode. Through the aforementioned mechanism, the designed biosensor achieves the transfer and separation of photoelectrons, utilizing the characteristics of TiO_2_ QDs and TiO_2_ NRs to enhance the efficiency of charge carriers and charge transfer. Meanwhile, the role of BDD protects the enzyme’s activity and improves the biocompatibility of the electrode.

Furthermore, TiO_2_ quantum dots (QDs) are zero-dimensional nanomaterials with small particle size, high catalytic activity, and large specific surface area [118]. They strongly absorb light and generate photoinduced electron–hole pairs, thereby enhancing the efficiency of PEC reactions. By growing TiO_2_ QDs in situ on TiO_2_ NRs, a structure that suppresses electron–hole recombination is formed, further enhancing the photoelectric response and photocatalytic activity [119]. This nanoscale interface structure achieves efficient transfer and utilization of photoelectrons, thereby enhancing the sensitivity and response capability of PEC biosensors. Considering the application requirements of enzyme biosensors, the loading of bioactive substances is crucial. BDD exhibits good biocompatibility, high bonding strength, and a wide range of adsorption, making it suitable for modifying photoactive materials and providing excellent carrier support [120]. By utilizing BDD-assisted visible light-excited PEC enzyme biosensor construction, efficient biosensing and photoelectric conversion can be achieved, providing reliable analytical tools for biomedical and environmental monitoring among other fields. Therefore, the combination of core–shell heterostructures, BDD and TiO_2_ QDs can be used to construct efficient 1D core/shell PEC enzyme biosensors (as shown in Figure 9a) [121,122]. These sensors leverage the advantages of interface effects, photoelectron transfer, and bio-carriers, exhibiting excellent photoelectric response capability and biocompatibility, and are expected to play a significant role in biosensing, photo electrochemistry, and biomedicine.

**Figure 10:**
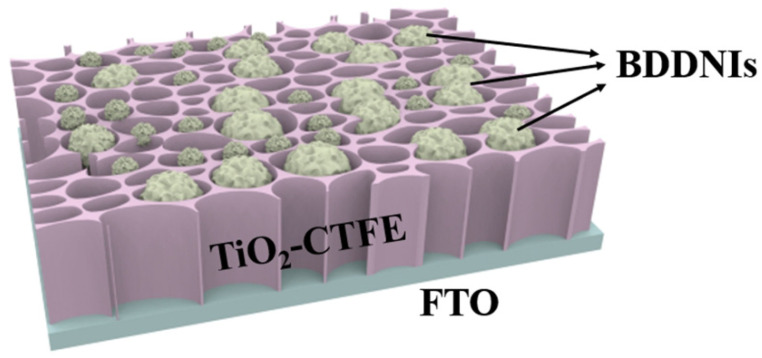
Schematic diagram of the TiO_2_-CTFE-BDDNIs sensing chip used to detect GM-derived exosomes [126].

Malignant glioma is a fatal brain cancer that is difficult to cure and prone to recurrence. Research has found that extracellular vesicles released by glioma cells contain the BIGH_3_ protein, which is associated with the malignant progression of glioma [125]. A technology called localized surface plasmon resonance (LSPR) biosensor can detect biomarkers in extracellular vesicles with high sensitivity and low cost. Xu et al. developed a novel LSPR biosensor called TiO_2_ Columnar Thin Film (CTF) combined with Au nano-islands (AuNIs) to detect extracellular vesicles released by glioma cells (GM) [126]. The key feature of this sensor is its enhancement effect, where a TiO_2_-CTF nano scaffold serves as the bottom layer, and AuNIs as the top layer, together forming a structure that enhances the local electric field. This structure provides the sensor with high sensitivity and selectivity in detecting extracellular vesicles [127]. By using the TiO_2_-CTF-AuNIs sensor, successful quantitative detection of the CD63 protein in extracellular vesicles derived from GM was achieved, demonstrating excellent detection performance with a low detection limit of 4.24 × 10^−3^ μg/mL. Additionally, they compared the TiO_2_-CTF-AuNIs sensor with a self-assembled monolayer gold nano-islands (SAM-AuNIs) LSPR biosensor. The results showed that the TiO_2_-CTF-AuNIs sensor exhibited more than 2.5 times higher sensitivity in CD63 detection compared to SAM-AuNIs, demonstrating superior sensing sensitivity performance. Transition metal oxide (such as Pt, Au, Pd, or Ag) modified TiO_2_ is a widely studied example, where one of the objectives of this structure is to significantly improve photocatalytic activity through charge separation, but its specific mechanism has not been fully revealed. In comparison, BDD has a wide electrochemical window and low electrode capacitance, making it perform exceptionally well in electrochemical sensors. BDD possesses excellent conductivity and chemical stability, allowing it to operate in various environments, including extreme pH values and high-temperature conditions [128]. Compared to noble metals, BDD has a wider light absorption capability, enabling sensor detection using a broader spectrum [129]. This makes the utilization of BDD-modified TiO_2_ as a sensor device more economical and feasible. Therefore, based on the research findings of Xu et al. [126], a TiO_2_-CTF-BDDNIs sensor (as shown in Figure 10) can be proposed. This novel TiO_2_-CTF-BDDNIs sensor may provide a promising tool for the early diagnosis and treatment of glioma, allowing doctors and researchers to have a more accurate understanding of the progression and malignancy of glioma, providing important information for personalized treatment and prognosis assessment [130].

The main limitation of TiO_2_-BDD sensors lies in selectivity: while high sensitivity and fast response are demonstrated, differentiation between gas species (e.g., CO vs. H_2_ vs. VOCs) remains weak. Furthermore, long-term drift due to surface contamination or vacancy passivation can degrade performance. Stability in real-world environments with humidity and temperature fluctuations is rarely validated.

## 3. Conclusions and Outlook

In summary, this article presents a rational design of TiO_2_-BDD heterostructures with various morphological structures, such as nano-layers, NPs, and NWs based on existing advanced optoelectronic device structures. TiO_2_-BDD heterostructures exhibit excellent performance characteristics including high sensitivity, low noise, high photoelectric conversion efficiency, and fast response speed which provides strong support for the development of future optoelectronic devices. In the field of detectors, TiO_2_-BDD heterostructures are ideal detector materials due to their high sensitivity and low noise characteristics that offer broad application prospects. These heterostructures demonstrate efficient and accurate detection and conversion of optical signals, making them applicable in areas such as optical communication, spectroscopic analysis, and biological imaging. In transistors field, the high electron mobility of BDD substrates enables TiO_2_-BDD heterostructures to have potential advantages in high-frequency power amplifiers and radio frequency switches. These heterostructures can achieve faster electron transport and higher operating frequencies in high-frequency electronic devices, potentially improving the performance of wireless communication and radar systems. For LEDs applications, the high photoelectric conversion efficiency and fast response speed of TiO_2_-BDD heterostructures make them important components of high-performance LEDs. These heterostructures can effectively capture and convert light energy, enhancing the efficiency and color purity of LEDs and promoting the development of energy-saving lighting and display technologies. Furthermore, TiO_2_-BDD heterostructures also show promising applications in the field of memory devices. Their use in non-volatile memory enables high-density data storage and fast read/write operations, which are of significant importance in information technology and big data processing.

As a new material combination, TiO_2_-BDD heterostructures have shown great potential in achieving excellent optoelectronic performance in current progress. Future research should focus on further optimizing the morphology and interface properties of these heterostructures. This includes exploring various nanoscale TiO_2_-BDD heterostructures, such as nano-layers, NPs, and NWs, as well as optimizing the interface quality between TiO_2_ and BDD. Fine interface engineering can maximize carrier transport efficiency, reduce recombination and trapping effects, and comprehensively improve device performance. TiO_2_-BDD heterostructures exhibit broad application prospects in detectors, LED, memory, FETs and sensing applications. Future research should further explore performance optimization strategies for these heterostructures in the aforementioned device categories, such as improving detection sensitivity and response speed, achieving high-frequency and high-power amplification, and enhancing light emission efficiency and color purity. Additionally, the application of TiO_2_-BDD heterostructures should be expanded to emerging fields such as memory devices and energy conversion to meet the demands of information technology and renewable energy devices. A key challenge for the practical application of TiO_2_-BDD heterostructures is their long-term stability and reliability. Future research should focus on evaluating the degradation mechanisms of these heterostructures and developing effective strategies such as surface passivation and encapsulation to improve the operational lifespan and reliability of devices. Furthermore, in-depth studies on performance stability under different application environments should be conducted to lay the foundation for the widespread commercialization of these heterostructures. The research on TiO_2_-BDD heterostructures requires close collaboration among multiple disciplines, including materials science, device physics, and process fabrication. In the future, interdisciplinary cooperation in these fields should be further promoted to systematically advance the design and innovation of TiO_2_-BDD heterostructures through means such as theoretical modeling, simulation analysis, and exploration of new processes. Additionally, stronger collaboration with the industry should be established to ensure timely translation and application of research findings.

To help visualize recent progress and identify existing performance gaps across different optoelectronic applications of TiO_2_-BDD heterojunctions, Table 1 summarizes key device-level metrics from representative studies. This comparative view reveals that while photodetector and memory performance is promising, LED and FET applications require further experimental validation, especially under extreme environmental conditions.

## Figures and Tables

**Table 1:** Summary of key performance metrics for TiO_2_-BDD and related heterojunction devices.

Application Type	Structure/Interface	Key Metrics	Value	Notes	Ref.
UV Photodetector	TiO_2_/BDD with Pd Schottky electrodes	Responsivity	Higher than pristine TiO_2_	Enhanced via gradient band and Schottky junction	[22]
		Dark current	250 fA @ 10 V	Very low, improves SNR	[23]
Gas Sensor (CO)	TiO_2_/BDD NSs	Response (*S*)	250% @500 ppm CO	Fast response and recovery time	[109]
		Response time	<10 s	Defined as “fast”	[109]
FET	TiO_2_ FET with BDD gate	*I_on_*/*I_off_* ratio	7.7 × 10^8^	Excellent switching	[89]
		Mobility (μeff)	5.74 cm^2^/V·s	High-performance oxide FET	[89]
Memory	TiO_2_/BDD/Ag NP sandwich	On/Off Ratio	~10^4^	Improved via trap-assisted tunneling	[77]
		Operation Voltage	~±1–2 V	SET/RESET controlled by Ag NPs traps	[77]
LED (Theoretical)	n-TiO_2_/p-BDD	Turn-on voltage	~3.2 V (est.)	No experimental data for full TiO_2_-BDD LED	[65]
		Emission range	3.2–5.5 eV	Theoretically covers visible range via defect states	[65]

## Data Availability

The data that support the findings of this study are available from the corresponding authors upon reasonable request.
